# Colorectal Cancer and the Human Gut Microbiome: Reproducibility with Whole-Genome Shotgun Sequencing

**DOI:** 10.1371/journal.pone.0155362

**Published:** 2016-05-12

**Authors:** Emily Vogtmann, Xing Hua, Georg Zeller, Shinichi Sunagawa, Anita Y. Voigt, Rajna Hercog, James J. Goedert, Jianxin Shi, Peer Bork, Rashmi Sinha

**Affiliations:** 1 Division of Cancer Epidemiology & Genetics, National Cancer Institute, Bethesda, Maryland, United States of America; 2 Division of Cancer Prevention, National Cancer Institute, Bethesda, Maryland, United States of America; 3 Structural and Computational Biology Unit, European Molecular Biology Laboratory, Heidelberg, Germany; 4 Department of Applied Tumor Biology, Institute of Pathology, University Hospital Heidelberg, Heidelberg, Germany; 5 Clinical Cooperation Unit Applied Tumor Biology, German Cancer Research Center (DKFZ), Heidelberg, Germany; 6 Molecular Medicine Partnership Unit (MMPU), University Hospital Heidelberg and European Molecular Biology Laboratory, Heidelberg, Germany; 7 Genomics Core Facility, European Molecular Biology Laboratory, Heidelberg, Germany; 8 Max Delbrück Centre for Molecular Medicine, Berlin, Germany; 9 Department of Bioinformatics Biocenter, University of Würzburg, Würzburg, Germany; Hospital for Sick Children, CANADA

## Abstract

Accumulating evidence indicates that the gut microbiota affects colorectal cancer development, but previous studies have varied in population, technical methods, and associations with cancer. Understanding these variations is needed for comparisons and for potential pooling across studies. Therefore, we performed whole-genome shotgun sequencing on fecal samples from 52 pre-treatment colorectal cancer cases and 52 matched controls from Washington, DC. We compared findings from a previously published 16S rRNA study to the metagenomics-derived taxonomy within the same population. In addition, metagenome-predicted genes, modules, and pathways in the Washington, DC cases and controls were compared to cases and controls recruited in France whose specimens were processed using the same platform. Associations between the presence of fecal Fusobacteria, *Fusobacterium*, and *Porphyromonas* with colorectal cancer detected by 16S rRNA were reproduced by metagenomics, whereas higher relative abundance of Clostridia in cancer cases based on 16S rRNA was merely borderline based on metagenomics. This demonstrated that within the same sample set, most, but not all taxonomic associations were seen with both methods. Considering significant cancer associations with the relative abundance of genes, modules, and pathways in a recently published French metagenomics dataset, statistically significant associations in the Washington, DC population were detected for four out of 10 genes, three out of nine modules, and seven out of 17 pathways. In total, colorectal cancer status in the Washington, DC study was associated with 39% of the metagenome-predicted genes, modules, and pathways identified in the French study. More within and between population comparisons are needed to identify sources of variation and disease associations that can be reproduced despite these variations. Future studies should have larger sample sizes or pool data across studies to have sufficient power to detect associations that are reproducible and significant after correction for multiple testing.

## Introduction

The human microbiome is the subject of a growing area of research since it is likely related to human health and disease. There is accumulating evidence that the microbiome plays a role in colorectal cancer (CRC) development or progression, potentially through inflammatory pathways or carcinogenic microbial metabolites [1], and microbial associations with CRC have been suggested in a number of studies [2–9]. For example, with next generation sequencing of the universal bacterial 16S rRNA gene in DNA extracted from feces, our group has shown that, compared to matched controls, CRC cases have lower community diversity, modestly lower relative abundance of Clostridia, and higher presence of *Fusobacterium* and *Porphyromonas* [2]. Of the previous microbiome and CRC studies, some used 16S rRNA gene sequencing [2, 4, 5, 7, 9], while others used whole-genome shotgun sequencing/shotgun metagenomics (WGSS; [3, 6, 8]). WGSS yields not only profiles of bacterial composition and diversity, but also estimates the functional potential of the microbiome [10].

We performed shotgun metagenomic sequencing of fecal samples from a CRC case-control study conducted in the 1980s in Washington, DC that were previously analyzed by 16S rRNA gene sequencing [2]. By subjecting the same samples to a different sequencing method, we were able to compare the previously observed 16S rRNA associations with data from shotgun metagenomic sequencing. In addition, by using this technology, we were able to investigate potential microbial gene-level associations with CRC which was not possible in the 16S rRNA gene sequencing data, and we compared gene-level associations with those detected in a previous French case-control study that applied the same metagenomics DNA extraction and sequencing platform and bioinformatics pipeline [8].

## Materials and Methods

### Primary study population

The fecal samples were collected in a CRC case-control study that has been previously described in detail [11] and the description of the analysis of the respective 16S rRNA gene sequencing study was previously published [2]. Briefly, CRC cases and frequency matched controls who were waiting surgery for non-oncological and non-gastrointestinal conditions were recruited from 1985 to 1987 in Washington DC, United States. Prior to surgery or other treatment, participants collected all stools over a two day period and stored them on dry ice. At the laboratory, the samples were freeze-dried, pooled, and stored continuously thereafter at -40°C. For the current shotgun metagenomic study, we selected samples from 52 cases and 52 controls (population WGSS DC). The cases and controls were matched by sex and body mass index (BMI; < 20 kg/m^2^ or ≥ 20 kg/m^2^). Associations in the WGSS DC analysis were compared to those in the previous 16S rRNA gene sequencing study (population 16S DC), which included 47 CRC cases and 94 control subjects from the same parent study. All 47 CRC cases from 16S DC were included in the WGSS DC and the 52 controls in WGSS DC were included in the 94 controls from 16S DC. Participants provided written informed consent and this study was approved by the Office of Human Subjects Research at the National Institutes of Health.

### Independent validation population

We included data from a previously published study (population F) as an independent validation set [8]. In brief, CRC cases and randomly chosen controls were recruited from 2004 to 2006 in Paris, France. Prior to colonoscopy, a fresh stool sample was collected and frozen at -20°C within four hours of collection. Population F included 53 CRC cases, 15 large adenoma cases, 27 small adenoma cases, and 61 normal controls. Since the controls from Washington DC may have also included undiagnosed small adenomas, the comparison case-control set from population F included 53 CRC cases and 88 controls (i.e., 27 small adenomas and 61 normal controls) and excluded the data from the 15 large adenomas.

We also included publically available shotgun metagenomic data from 292 MetaHIT participants [12, 13] and 94 Human Microbiome Project (HMP) Phase I participants [14] for comparison of overall diversity, richness, and evenness with our samples.

### DNA extraction and whole-genome shotgun sequencing

The freeze-dried fecal samples from WGSS DC were defrosted, resuspended in phosphate-buffered saline, and an aliquot was shipped to the Genomics Core Facility, European Molecular Biology Laboratory in Heidelberg, Germany on dry ice. The methods for DNA extraction, library preparation, and whole-genome shotgun sequencing have been described in detail [8] and were the same for population WGSS DC and population F. In brief, DNA was extracted from the fecal samples using the GNOME DNA Isolation Kit (MP Biomedicals) with minor modifications. Whole-genome shotgun sequencing of the extracted DNA was conducted using the Illumina HiSeq 2000/2500 (Illumina, San Diego, USA). The samples were sequenced with a 100-bp read length for paired-end sequences at the Genomics Core Facility, European Molecular Biology Laboratory, Heidelberg, Germany with a targeted sequencing depth of 5 Gbp.

### Bioinformatics

The general strategy for the bioinformatic processing of the whole-genome sequencing data has been previously described in detail [8] and was the same for both WGSS DC and population F. Taxonomic abundance profiles summarized at NCBI taxonomic ranks ranging from species to phylum and metagenomic operational taxonomic units (mOTU) [15, 16] were created using MOCAT [17]. MOCAT was also used to functionally annotate genes extracted from metagenomic assemblies to the KEGG database (version 62) [18]. Ecological indices (Shannon diversity, species richness, and community evenness) were calculated based on mOTU relative abundances and downsampled to 2000 inserts using the vegan R software package [19]. One participant from population WGSS DC and four participants from population F were excluded due to lower read coverage.

### Statistical analysis

We compared Shannon diversity, richness, and evenness for population WGSS DC, population F, MetaHIT, and HMP Phase I samples and tested case-control differences in population WGSS DC and population F using the Kruskal Wallis test. Then, for both the primary study population (population WGSS DC: 52 cases vs 52 controls) and the independent validation population (population F: 53 cases vs 88 controls), we tested for the associations between case/control status and both the relative abundance and presence/absence of the different taxonomic levels and gene categories (i.e., genes, modules, and pathways). A logistic regression model with adjustment for age, sex, and body mass index (BMI) was used and the p values were calculated based on the Wald test (S1 Table). Three CRC cases from population WGSS DC were missing BMI data so we included these values using sex-specific means of the CRC cases. For comparability with the 16S DC study, we also calculated an unadjusted logistic regression model for a two-sided Wald chi-squared test and a two-sided non-parametric Wilcoxon test for presence/absence and relative abundance of specific taxa, respectively. We generated QQ plots of the—log(observed p value) versus the—log(p values under a normal distribution) within WGSS DC and population F for all taxonomic levels and gene categories to ascertain potentially statistically significant associations after correction for multiple comparisons. For the gene category data in population F, we used Bonferroni correction of the p value to determine statistical significance (i.e., p < 0.05/number of tests) and considered a p value < 0.05 to be statistically significant for reproducibility analyses in WGSS DC. All statistical analyses were conducted using R (version 3.0.0).

## Results

Characteristics of the 52 CRC cases and 52 controls from population WGSS DC are presented in Table 1. They were well matched by sex and BMI. However, CRC cases had a higher proportion of non-Hispanic blacks (23.1% in cases and 5.8% in controls), lower education level (15.4% of cases and 3.8% of controls had less than a high school education), and more current smokers (13.5% of cases and 3.8% of controls). Within the CRC cases, 28.8% of cases had cancer in the right colon and 34.6% had cancer in the left colon. The majority of CRCs were invasive with no known metastases (40.4%), but 34.6% were metastatic.

**Table 1:** Descriptive characteristics of the colorectal cancer cases and controls (population WGSS DC), Washington DC, USA, 1985–1987.

	Cases		Controls	
	N = 52		N = 52	
	N/Mean	%/SD	N/Mean	%/SD
**Sex**				
Male	37	71.2%	37	71.2%
Female	15	28.8%	15	28.8%
**Age**	61.8	13.6	61.2	11.0
**Race**				
Non-Hispanic white	39	75.0%	47	90.4%
Non-Hispanic black	12	23.1%	3	5.8%
Other	1	1.9%	2	3.8%
**Education**				
Less than high school	8	15.4%	2	3.8%
High school graduate	11	21.2%	10	19.2%
1–3 years of college/graduate	10	19.2%	9	17.3%
4–5 years of college/graduate	12	23.1%	15	28.8%
6+ years of college/graduate	8	15.4%	16	30.8%
Missing data	3	5.8%	0	0.0%
**Smoking history**				
Never	24	46.2%	22	42.3%
Former	18	34.6%	28	53.8%
Current	7	13.5%	2	3.8%
Missing data	3	5.8%	0	0.0%
**Body mass index**	24.9	4.2	25.3	4.3
**Alcohol (drinks/wk)**	7.4	11.9	6.1	10.4
**Cancer site**				
Right colon	15	28.8%	NA	
Left colon	18	34.6%	NA	
Rectal	14	26.9%	NA	
Missing data	5	9.6%	NA	
**Cancer stage**			NA	
Pre-invasive	12	23.1%	NA	
Invasive, no known metastases	21	40.4%	NA	
Known metastases	18	34.6%	NA	
Missing data	1	1.9%	NA	

NA: Not applicable

### Colorectal cancer associations in the WGSS DC versus 16S DC

In the previous 16S rRNA gene sequencing analysis in this population (16S DC), the presence of 4 taxa and the relative abundance of 3 taxa were significantly associated with CRC case status with false discovery rate-adjusted p values less than 0.05. As seen in Table 2, we reproduced a significant association between the presence of the Fusobacteria phyla and CRC case status (p = 0.003), specifically that 76.9% of cases and 48.1% of controls had detectable Fusobacteria. This reproduces the association of Fusobacteria with case status in the 16S DC analysis; although detection was lower (36.2% of cases and 16.0% of controls, Table 2). Compared to the 16S DC, the WGSS also had higher prevalent detection rate for other taxa, and it reproduced a significant association between the presence of *Fusobacterium* (p = 0.006) and *Porphyromonas* (p = 0.032) with CRC case status. The association between *Atopobium* and CRC from the 16S DC was not reproduced in the WGSS (Table 2). As seen in Table 3, we did not reproduce associations between the relative abundance of specific taxa and CRC case status, although the association between the relative abundance of Clostridia tended to be lower in cases (p = 0.092). Notably, relative abundance of Clostridia estimated in the WGSS was two-fold lower for both cases and controls compared to that in the 16S DC study. In population WGSS DC, the class with a highest relative abundance was Bacteroidia, which had a relative abundance of 53.2% in cases and 50.9% in controls (S1 Table).

**Table 2:** Comparison of significant taxa detected in 16S rRNA gene sequencing data with whole-genome shotgun sequencing data (presence/absence of taxa).

	Population 16S DC			Population WGSS DC		
	Case	Control		Case	Control	
Taxa (phylum; class; order; family; genus)	%	%	P^1^	%	%	P^1^
Fusobacteria (phylum)	36.2	16.0	0.007	76.9	48.1	0.003
Fusobacteria;Fusobacteria;Fusobacteriales;Fusobacteriaceae;Fusobacterium	31.9	11.7	0.004	75.0	48.1	0.006
Actinobacteria;Actinobacteria;Coriobacteriales;Coriobacteriaceae;Atopobium	19.2	2.1	<0.001	53.8	44.2	0.328
Bacteroidetes;Bacteroidia;Bacteriodales;Porphyromonadaceae;Porphyromonas	27.7	7.5	0.001	61.5	40.4	0.032

^1^ P value based on two-sided chi-squared test

**Table 3:** Comparison of significant relative abundance of taxa detected in 16S rRNA gene sequencing data with whole-genome shotgun sequencing data.

	Population 16S DC			Population WGSS DC		
	Case	Control		Case	Control	
Taxa (phylum; class; order; family; genus)	%	%	P^1^	%	%	P^1^
Firmicutes;Clostridia (class)	68.6	77.8	0.005	33.9	39.0	0.092
Firmicutes;Clostridia;Clostridiales;Lachnospiraceae;Coprococcus	1.7	3.7	0.002	1.2	1.4	0.977
Firmicutes;Clostridia;Clostridiales;Lachnospiraceae;Other	16.1	21.2	0.005	NA^2^	NA^2^	NA^2^

^1^ P value based on two-sided non-parametric Wilcoxon test

^2^ It was not possible to estimate the “other” genus using whole-genome shotgun metagenomics

### Colorectal cancer associations in the WGSS DC versus Population F

**Fig 1:**
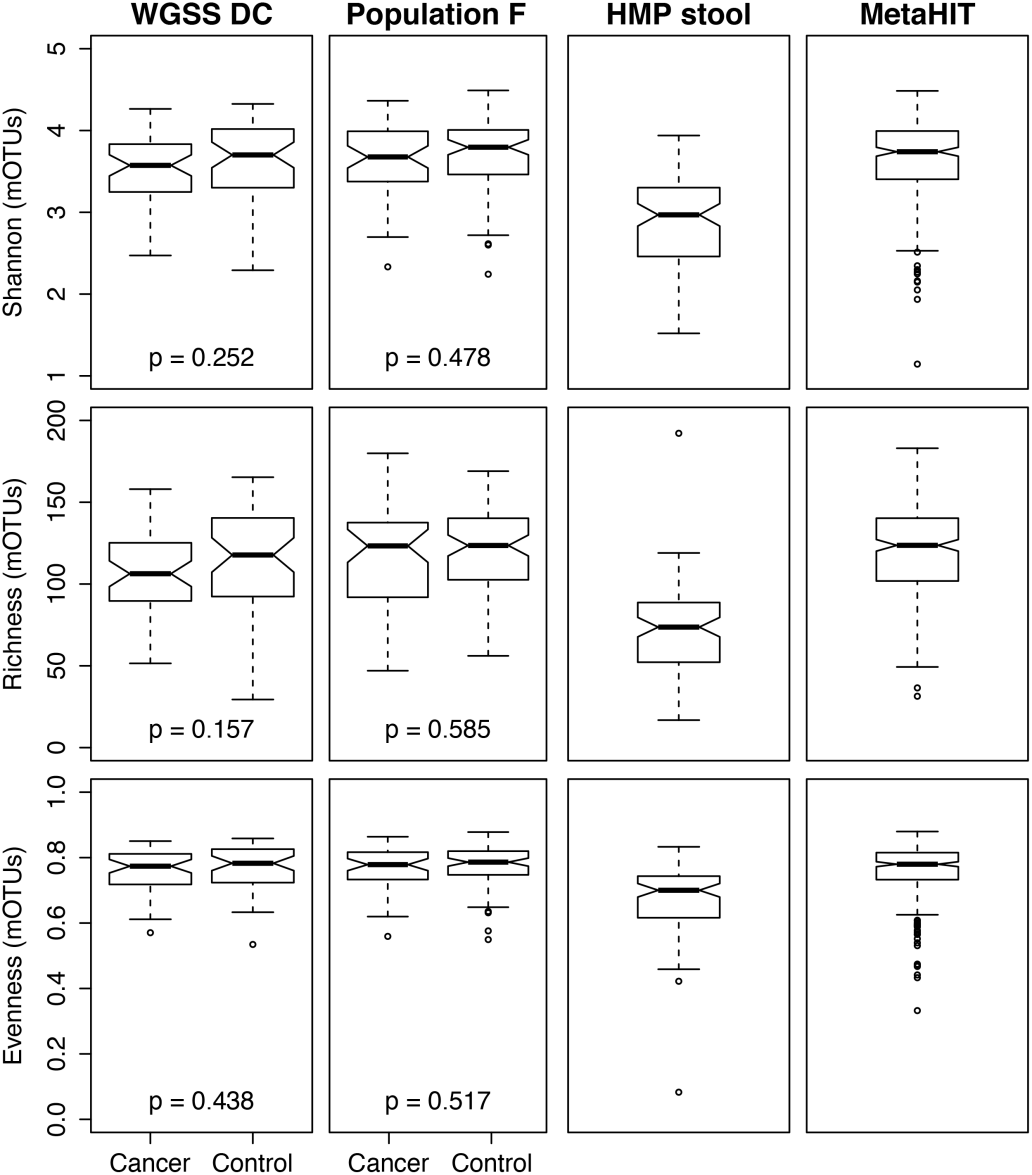
Comparison of Shannon diversity index, species richness, and community evenness for fecal samples from the Human Microbiome Project (HMP) Phase I (N = 94), MetaHIT (N = 292), and colorectal cancer cases and controls from population WGSS DC and population F. Statistical differences between colorectal cancer cases and controls were tested using the Kruskal-Wallis test.

In population WGSS DC, there were no significant differences between CRC cases and controls for Shannon diversity, richness, or evenness based on mOTUs, although in general the controls had slightly higher alpha diversity compared to cases (Fig 1). Shannon diversity, richness, and evenness were similar for population WGSS DC, population F, and the MetaHIT samples, whereas the HMP samples tended to have slightly lower Shannon diversity, richness, and evenness.

**Fig 2:**
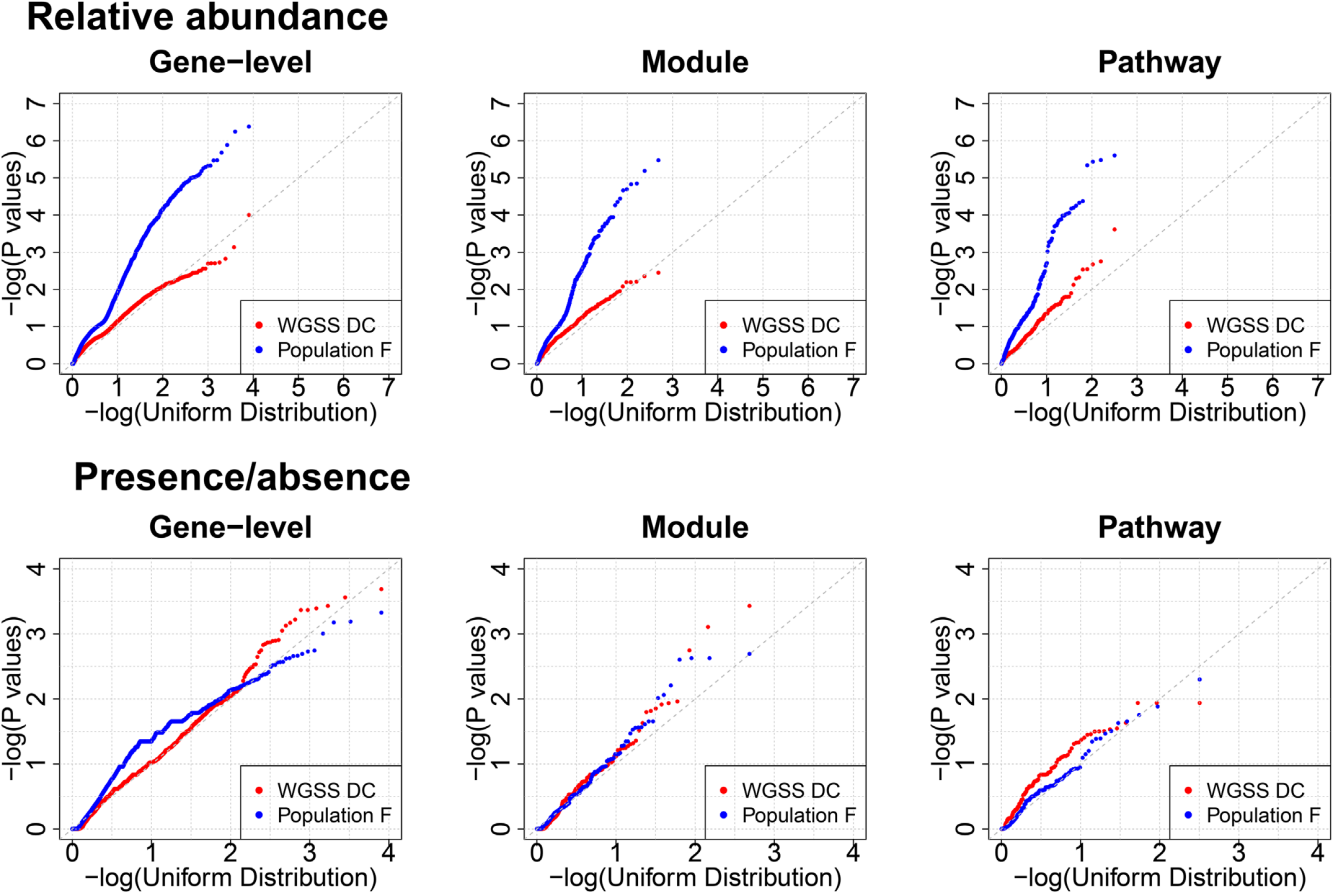
QQ plot of p values for the association between the relative abundance (top) and presence/absence (bottom) of KEGG genes, modules, and pathways with colorectal cancer case status from fecal samples from population WGSS DC and population F.

CRC case status in population F [8] was strongly associated with the relative abundance of many metagenome-derived KEGG genes, modules, and pathways (as seen by the strong deviation from the 45° degree line in Fig 2), but this was not seen in the WGSS DC population. For the presence of KEGG genes, modules, and pathways, there was little evidence for any associations with CRC case status in either study population (Fig 2). Since associations were detected for the relative abundance of the gene-level data only in population F, we attempted to reproduce statistically significant associations after Bonferroni correction in population F with the WGSS DC data without correction for multiple comparisons.

In contrast to the global assessment (Fig 2), when we considered the significant associations between the relative abundance of KEGG genes (p < 0.05/8028), modules (p < 0.05/485), and pathways (p < 0.05/318) within population F, the cancer associations were reproduced (p < 0.05) in the WGSS DC for four of 10 genes: aminomethyltransferase (K00605), tryptophanase (K01667), peptide methionine sulfoxide reductase msrA/msrB (K12267), and putative membrane protein (K01421) (Table 4). Likewise, the WGSS DC reproduced cancer associations for three of nine modules: leucine degradation, leucine = > acetoacetate + acetyl-CoA (M00036), citrate cycle, second carbon oxidation, 2-oxoglutarate = > oxaloacetate (M00011), and methionine biosynthesis, apartate = > homoserine = > methionine (M00017) (Table 5). Out of the 17 statistically significant pathway associations in population F, the WGSS DC reproduced associations with seven pathways: citrate cycle (ko00020), lipoic acid metabolism (ko00785), valine, leucine, and isoleucine degradation (ko00280), amyotrophic lateral sclerosis (ko05014), lysine biosynthesis (ko00300), geraniol degradation (ko00281), and nitrogen metabolism (ko00910) (Table 6). No additional significant associations with CRC were found in the WGSS DC.

**Table 4:** Statistically significant associations after Bonferroni correction (p < 0.05/8028) between the relative abundance of a gene and colorectal cancer case status from population F and observed associations from population WGSS DC.

	Population F			Population WGSS DC		
	Case	Control	P^1^	Case	Control	P^1^
**K00605**	**0.034%**	**0.021%**	**4.15E-07**	**0.037%**	**0.032%**	**3.00E-02**
K07173	0.045%	0.056%	5.64E-07	0.045%	0.047%	4.18E-01
**K01667**	**0.020%**	**0.010%**	**1.29E-06**	**0.021%**	**0.016%**	**1.20E-02**
**K12267**	**0.030%**	**0.019%**	**2.08E-06**	**0.031%**	**0.026%**	**2.74E-02**
K00177	0.038%	0.025%	3.36E-06	0.040%	0.037%	1.10E-01
**K01421**	**0.068%**	**0.101%**	**3.36E-06**	**0.065%**	**0.075%**	**3.91E-02**
K01586	0.081%	0.092%	4.67E-06	0.087%	0.091%	8.39E-02
K00176	0.031%	0.020%	4.75E-06	0.029%	0.027%	3.22E-01
K01963	0.036%	0.048%	5.04E-06	0.036%	0.039%	9.38E-02
K00394	0.011%	0.019%	5.51E-06	0.010%	0.011%	5.98E-01

Note: Genes in bold were reproduced (p < 0.05) in population WGSS DC

^1^ P value based on two-sided Wald chi-squared test after adjustment for age, sex, and body mass index

**Table 5:** Statistically significant associations after Bonferroni correction (p < 0.05/485) between the relative abundance of a module and colorectal cancer case status from population F and observed associations from population WGSS DC.

	Population F			Population WGSS DC		
	Case	Control	P^1^	Case	Control	P^1^
M00311	0.342%	0.226%	3.32E-06	0.376%	0.349%	1.45E-01
**M00036**	**0.266%**	**0.221%**	**6.42E-06**	**0.270%**	**0.253%**	**2.14E-02**
**M00011**	**1.056%**	**0.834%**	**1.40E-05**	**1.157%**	**1.087%**	**4.51E-02**
M00045	0.163%	0.096%	1.48E-05	0.198%	0.174%	5.78E-02
**M00017**	**0.781%**	**0.841%**	**2.03E-05**	**0.784%**	**0.807%**	**2.32E-02**
M00185	0.095%	0.147%	2.16E-05	0.081%	0.081%	9.67E-01
M00144	0.646%	0.483%	3.57E-05	0.727%	0.672%	8.97E-02
M00373	0.276%	0.222%	4.42E-05	0.298%	0.280%	6.24E-02
M00173	1.721%	1.519%	5.44E-05	1.815%	1.783%	3.72E-01

Note: Modules in bold were reproduced (p < 0.05) in population WGSS DC

^1^ P value based on two-sided Wald chi-squared test after adjustment for age, sex, and body mass index

**Table 6:** Statistically significant associations after Bonferroni correction (p < 0.05/318) between the relative abundance of a pathway and colorectal cancer case status from population F and observed associations from population WGSS DC.

	Population F			Population WGSS DC		
	Case	Control	P^1^	Case	Control	P^1^
**ko00020**	**1.083%**	**0.944%**	**2.48E-06**	**1.103%**	**1.064%**	**3.54E-02**
**ko00785**	**0.071%**	**0.045%**	**3.31E-06**	**0.075%**	**0.065%**	**2.12E-02**
**ko00280**	**0.385%**	**0.323%**	**3.65E-06**	**0.382%**	**0.358%**	**2.91E-03**
ko04964	0.052%	0.035%	4.56E-06	0.052%	0.048%	1.70E-01
ko00400	1.245%	1.335%	4.22E-05	1.239%	1.258%	2.08E-01
ko00430	0.245%	0.226%	4.68E-05	0.248%	0.244%	3.23E-01
ko00195	0.615%	0.717%	5.53E-05	0.640%	0.669%	1.44E-01
ko00627	0.162%	0.134%	6.29E-05	0.159%	0.150%	9.51E-02
**ko05014**	**0.019%**	**0.011%**	**6.71E-05**	**0.020%**	**0.016%**	**2.60E-02**
**ko00300**	**1.043%**	**1.102%**	**8.74E-05**	**1.039%**	**1.064%**	**4.76E-02**
ko00983	0.579%	0.548%	9.11E-05	0.588%	0.589%	8.33E-01
**ko00281**	**0.050%**	**0.030%**	**9.71E-05**	**0.058%**	**0.049%**	**4.42E-02**
**ko00910**	**1.613%**	**1.448%**	**1.03E-04**	**1.680%**	**1.622%**	**3.51E-02**
ko00360	0.396%	0.352%	1.04E-04	0.395%	0.385%	2.58E-01
ko00270	1.736%	1.827%	1.29E-04	1.703%	1.730%	1.65E-01
ko00643	0.027%	0.017%	1.33E-04	0.021%	0.020%	4.31E-01
ko00720	1.622%	1.514%	1.42E-04	1.663%	1.645%	3.71E-01

Note: Pathways in bold were reproduced (p < 0.05) in population WGSS DC

^1^ P value based on two-sided Wald chi-squared test after adjustment for age, sex, and body mass index

## Discussion

This study had two primary aims: 1) to compare the previously observed 16S rRNA gene associations with data from whole-genome shotgun metagenomic sequencing; and 2) to investigate potential microbial gene-level associations with CRC in different populations. For the first aim, the metagenomics approach reproduced some of the previously observed associations in the 16S rRNA gene analysis, most notably higher likelihood of detecting taxa in the Fusobacteria phylum and *Fusobacterium* genus among CRC cases. One large difference between the two studies was the sensitivity for detecting taxa. For example, the Fusobacteria phyla was detected in 36.2% of the cases and 16.0% of the controls in the 16S rRNA gene study, but using whole-genome shotgun metagenomics, Fusobacteria was detected in 76.9% of the cases and 48.1% of the controls. It is unclear what may cause the differences between the 16S rRNA and WGSS results, but it may be due to the 16S rRNA gene variable region sequenced, the depth of sequencing, the bioinformatic assignment of taxonomy, or technical differences. These variations in detecting the presence and relative abundance of taxa demonstrate an important difference when comparing 16S rRNA sequencing to whole-genome shotgun metagenomic studies and should be studied in more detail in the future.

For our second aim, the WGSS DC did reproduce some of the specific, statistically significant genes, modules, and pathways detected in population F with CRC case status [8]. Two related modules and pathways were identified in independent models: M00011 (Citrate cycle, second carbon oxidation, 2-oxoglutarate = > oxaloacetate) and ko00020 (citrate cycle/TCA cycle); and M00036 (leucine degradation, leucine = > acetoacetate + acetyl-CoA) and ko00280 (valine, leucine, and isoleucine degradation). It is possible that these, and other functional capabilities are related to CRC, but further studies are needed. Shannon diversity, richness, and evenness based on whole-genome shotgun metagenomics were not associated with CRC case status in the WGSS DC, but these estimates were similar to those in MetaHIT and population F.

Our reproducibility of statistically significant associations from a previous study [8] provides important information about future data pooling given the large differences between these two sets of data. Our samples were collected in the 1980s in the United States while the samples for population F were collected in the 2000s in France. There is some evidence that storage of fecal samples at low temperatures maintains the microbial community structure [20, 21], however, to our knowledge, this has not been tested for samples stored for almost 30 years. And given previous work that suggests that microbial associations with Type 2 diabetes may differ by population [22, 23], although these differences may be driven by metformin use [24], it is encouraging that some of the associations were robust between populations in the United States and France from different years. Additionally, the fecal samples in our study were collected prior to hospitalization and treatment from all bowel movements over the course of two days and then freeze-dried. This contrasts with the methods for population F, where samples were collected 2 weeks to 3 days before colonoscopy, but always prior to bowel cleansing, and were an aliquot from one bowel movement which was frozen within four hours. Freeze-drying of fecal samples has been found to potentially affect the relative abundances of different taxa for infant fecal samples [25], so it is reassuring to replicate some findings between different storage methods. In addition, the WGSS samples appeared to have similar diversity measures compared to another shotgun metagenomic study, MetaHIT, which also included a different population and collections. As has been seen in human genome-wide association studies, large sample sizes are needed to detect associations that survive correction for multiple testing. With these differences in time period of collection, population, and sample collection, the similarities in associations between microbial taxonomic and gene-level data with CRC case status provides some support for the pooling of data across heterogeneous studies. Additional work has been conducted to assess the ideal collection methods for future fecal collections [26–30] and the effect of laboratory handling procedures and bioinformatic processing of the data [31] that can provide additional information for downstream data pooling or meta-analysis.

Other previous studies have investigated associations between the fecal microbiome and CRC [32]. Similar to our findings, a number of studies did not detect an overall difference between CRC cases and controls for measures of community diversity [4, 5, 9]. However, one study observed that CRC cases had increased gene and genus richness compared to controls [3], while another study detected reduced gene richness and gene alpha diversity in CRC cases compared to controls, although the association was not statistically significant after adjustment for fecal sample collection after colonoscopy [6]. In agreement with our findings, most previous studies found that CRC cases were more likely to have detectable or higher levels of *Fusobacterium* compared to controls [3, 5–7, 9], while only some studies detected higher levels or detection of *Porphyromonas* in CRC cases compared to controls [3, 5, 7]. In a previous whole-genome shotgun metagenomic study, the module M00036 and KEGG pathways ko00280 and ko00910 were found to be significantly enriched in CRC cases compared to controls [6] similar to what was detected in this study. In the other previous whole-genome shotgun metagenomic study, our findings for KEGG pathways ko00020, ko00280, ko00281, ko00300, ko00785 were confirmed, but an association for KEGG pathway ko00910 was in the opposite direction from what we observed [3]. In summary, we confirmed some associations observed in previous research, but all previous studies (16S and whole-genome shotgun sequencing) had low power. Furthermore, these previous studies may not have been able to adequately adjust for potential confounders, which could explain some of the variability between studies. Due to the multiple comparisons in microbiome analyses, data pooling will be critical to overcoming the limited power in these analyses.

The current study is not without limitations. First, all of the fecal samples were collected cross-sectionally, so it is not possible to determine if the microbial changes occurred prior to cancer development or if they were due to the development of cancer. In addition, this study had a relatively small sample size and therefore, we were underpowered to detect many statistically significant associations after correction for multiple testing. Finally, our healthy controls were hospital based controls awaiting elective surgery and may not represent the general population at that time. However, our study also has strengths. We were able to leverage existing sample resources that were collected over 30 years ago and to reproduce associations with CRC from a current study. Our fecal sample was from a two day collection which may be more representative of the typical gut microbiome. We also were able to utilize other existing data sources for comparison.

In this study, we were able to use whole-genome shotgun metagenomic sequencing to reproduce a number of significant findings in the same population that was assessed using 16S rRNA gene sequencing [2]. The current study also reproduced some significant gene-level associations with CRC from a previous whole-genome shotgun metagenomic study of patients in France [8]. Future studies pooling data across time, population, and sample collection method will help overcome some of the statistical power issues facing epidemiologic studies of the microbiome and will be key to identifying important associations that may be involved in CRC detection or prevention. In addition, since all current studies are cross-sectional, it is imperative that prospective cohort studies include a fecal sample collection in order to study the effect of the human gut microbiome on adverse health outcomes, like CRC.

## Supporting Information

S1 Table Average relative abundance or detection of taxonomic assignments (i.e., phylum, class, order, family, genus, species, SpecI, Motu) and gene categories (i.e., gene, module, and pathway) for population WGSS DC and population F. Each tab represents a specific taxonomic level or gene assignment. The mean relative abundance or average detection (presence/absence) is presented for cases and controls, and the p value from a Wald test adjusting for age, sex and body mass index (BMI) is provided.
(XLS)

## Abbreviations

BMI: Body mass index
CRC: Colorectal cancer
DC: District of Columbia
HMP: Human Microbiome Project
mOTU: Metagenomic operational taxonomic unit
OTU: Operational taxonomic unit
WGSS: Whole-genome shotgun sequencing
